# Myosin Light Chain Kinase (MLCK) Gene Influences Exercise Induced Muscle Damage during a Competitive Marathon

**DOI:** 10.1371/journal.pone.0160053

**Published:** 2016-08-02

**Authors:** Juan Del Coso, Marjorie Valero, Beatriz Lara, Juan José Salinero, César Gallo-Salazar, Francisco Areces

**Affiliations:** Exercise Physiology Laboratory, Camilo José Cela University, Madrid, Spain; University of Chicago, UNITED STATES

## Abstract

Myosin light chain kinase (MLCK) phosphorylates the regulatory light chain (RLC) of myosin producing increases in force development during skeletal muscle contraction. It has been suggested that MLCK gene polymorphisms might alter RLC phosphorylation thereby decreasing the ability to produce force and to resist strain during voluntary muscle contractions. Thus, the genetic variations in the MLCK gene might predispose some individuals to higher values of muscle damage during exercise, especially during endurance competitions. The aim of this investigation was to determine the influence of MLCK genetic variants on exercise-induced muscle damage produced during a marathon. Sixty-seven experienced runners competed in a marathon race. The MLCK genotype (C37885A) of these marathoners was determined. Before and after the race, a sample of venous blood was obtained to assess changes in serum myoglobin concentrations and leg muscle power changes were measured during a countermovement jump. Self-reported leg muscle pain and fatigue were determined by questionnaires. A total of 59 marathoners (88.1%) were CC homozygotes and 8 marathoners (11.9%) were CA heterozygotes. The two groups of participants completed the race with a similar time (228 ± 33 vs 234 ± 39 min; *P* = 0.30) and similar self-reported values for fatigue (15 ± 2 vs 16 ± 2 A.U.; *P* = 0.21) and lower-limb muscle pain (6.2 ± 1.7 vs 6.6 ± 1.8 cm; *P* = 0.29). However, CC marathoners presented higher serum myoglobin concentrations (739 ± 792 vs 348 ± 144 μg·mL^-1^; *P* = 0.03) and greater pre-to-post- race leg muscle power reduction (-32.7 ± 15.7 vs -21.2 ± 21.6%; *P* = 0.05) than CA marathoners. CA heterozygotes for MLCK C37885A might present higher exercise-induced muscle damage after a marathon competition than CC counterparts.

## Introduction

During skeletal muscle contraction, two small protein subunits, the essential light chain and phosphorylatable regulatory light chain (RLC) of myosin facilitate the formation of cross-bridges between the sarcomeric proteins actin and myosin. Although the binding of Ca^2+^ to tropomyosin-troponin is the primary regulator of skeletal muscle contraction, RLC plays an essential modulatory role in force development [1]. Specifically, the phosphorylation of the RLC in skeletal muscle fibers increases isometric force and the rate of force production at submaximal and maximal levels of calcium activation [2, 3]. The Ca^2+^/calmodulin dependent myosin light-chain kinase, also known as MLCK, is responsible for the phosphorylation of RLC predominantly in type II fibers [4] and thus MLCK functioning can affect muscle force production and/or the ability to resist muscle strain [5].

To date, only two investigations have analyzed the role of MLCK gene polymorphisms during *in vivo* skeletal muscle contractions in humans. Clarkson, Hoffman (5] hypothesized that individuals with a specific single nucleotide polymorphism (SNP) in the MLCK gene would present greater levels of exercise-induced muscle damage. In this investigation, the genetic variants of two SNPs of the MLCK gene (C49T and C37885A) were associated with a loss of muscle strength and increased blood markers of muscle damage (myoglobin and creatine kinase) 4 days after a protocol of 50 maximal eccentric contractions of the elbow flexor muscles. They found that those individuals who were CA heterozygotes for C37885A had a significantly greater strength loss and greater increase in creatine kinase than CC homozygotes. Besides, individuals who were TT homozygotes for C49T had a significantly greater increase in creatine kinase and myoglobin after exercise when compared with the CT and CC individuals. Deuster, Contreras-Sesvold (6] investigated these same SNPs in individuals that had suffered hospitalization or two severe episodes of exertional rhabdomyolysis, a clinical condition related to the breakdown of skeletal muscle from extreme physical exertion. They found that the A allele for C37885A was significantly more frequent in individuals with a history of clinical exertional rhabdomyolysis, while C49T had no influence on this condition.

However, in these two pioneer investigations, the influence of the MCLK genotype on the ability of skeletal muscle to resist muscle strain thru RLC phosphorylation was investigated using a local muscle activity (elbow muscle flexors) that does not replicate any exercise or sport activity [5] or using clinical episodes of exertional rhabdomyolysis that could be related to excessive or unbearable exercise protocols rather than a genetic cause [6]. Thus, the aim of the current investigation was to determine the influence of the MLCK genetic variants on exercise-induced muscle damage during a whole-body and real exercise activity (a real marathon competition) in order to increase the validity and applicability of the outcomes. We hypothesized that marathoners who were carriers of the A allele for MLCK C37885A would present higher signs of exercise-induced muscle damage, at least when compared to CC homozygotes.

## Methods

### Ethics statement

All participants provided written informed consent, subsequent to receiving both written and verbal information regarding the nature of this investigation. The study was approved by the Camilo Jose Cela University Ethics Committee in accordance with the latest version of the Declaration of Helsinki.

### Subjects

Sixty-seven healthy and experienced marathon runners volunteered to participate in this study (Table 1). Inclusion criteria were as follows: age between 18 and 65 years, being free of any history of chronic muscle, cardiac or kidney disorders, participating in the marathon at maximal intensity, and having a running experience of at least 3 years. Exclusion criteria were as follows: taking medications during the two weeks prior to competing or having suffered a musculoskeletal injury in the 3 months previous to the competition.

**Table 1:** Age, anthropometric characteristics, running experience, best race time in the marathon and training status of marathoners with different MLCK genotypes (1 SNP tested C37885A). Data are mean ± SD for CC homozygotes and for CA heterozygotes.

*Variable (units)*	CC	CA	*p*
n	59	8	-
Men/women	53/6	7/1	-
Frequency (%)	88.1	11.9	-
Age (yr)	42.4 ± 8.7	40.7 ± 9.4	0.31
Body mass (kg)	72.3 ± 10.2	75.3 ± 17.1	0.25
Body height (cm)	175 ± 8	172 ± 7	0.22
Running experience (yr)	12.0 ± 9.3	7.9 ± 5.4	0.13
Best race time in the marathon (min)	216 ± 33	210 ± 31	0.35
Completed marathons (number)	8.2 ± 11.5	9.4 ± 15.9	0.40
Average training distance /week (km)	58.9 ± 23.6	64.4 ± 26.1	0.27
Training sessions /week (number)	4.5 ± 1.0	4.3 ± 1.2	0.24

### Experimental design

All the participants underwent the same testing under the same experimental conditions. Participants completed the 2015 edition of the Rock’n’Roll Madrid Marathon with no indications about running pace or fluid and food strategies. Before and after the race, typical variables for the determination of the level of exercise-induced muscle damage (leakage of intracellular myoglobin into systemic circulation, muscle pain and muscle force and power reduction) were measured in all participants. Afterwards, the pre-competition blood sample was used to determine the MLCK genotype using the SNP C37885A and the participants were divided into two groups: CC homozygotes and CA heterozygotes. There was no participant with the genotype AA for this SNP in the study sample. The proportion of men and women in each group was very similar and thus, we have analyzed all the data without considering the sex of the individuals.

### Experimental protocol

The day before the race, a blood sample was obtained from an antecubital vein after 10 min of supine resting. The blood was allowed to clot and serum was separated by centrifugation (10 min at 5000 g) and frozen at -80°C until the day of analysis. At a later date, the serum portion was analyzed for creatine kinase and myoglobin concentrations by means of an autoanalyzer (Access II, Beckman-Coulter Instruments, USA). Then, participants underwent a standardized 10-min warm-up and performed two maximal countermovement vertical jumps on a force platform (Quattrojump, Kistler, Switzerland). The pre-race jumps were performed with the competition clothes and shoes and the attempts were separated by 1 min of rest. In each jump, maximal leg power output during the concentric phase of the jump was determined from ground reaction forces. The highest jump was used for statistical analysis.

The day of the race, participants had their usual pre-competition meal at least 3 h before the race and adopted their typical pre-race routines. Participants completed the race (13.0 ± 1.0°C dry temperature and 88 ± 1% of relative humidity) at their own pace and drank *ad libitum* at the hydration stations placed at 5-km intervals. During the race, participants wore a race bib with a time-chip to calculate the actual amount of time that it took them from the start line of the race to the finish line (net time). Within 2 min of the end of the marathon race, participants went to a finish area where they performed two countermovement vertical jumps, as described above. Participants then rested for 5 min and a venous blood sample was obtained. During this time, lower-limb muscle pain was measured using a 10-cm visual analog scale where participants self-rated the score from 0 (no muscle pain at all) to 10 cm (unbearable muscle pain). The rating of perceived exertion was also assessed using the Borg scale [7].

### Genetic testing

Genomic DNA was isolated from the whole blood obtained before the race (QIAamp^®^ DNA Blood Mini Kit, QIAGEN, The Netherlands) according to the manufacturer’s protocol. MLCK C37885A (c.62C>A, p.P21H, rs28497577) genotyping was performed using a TaqMan^®^ SNP genotyping assay (Life Technologies™, USA) that employs the 5’ nuclease activity of Taq DNA polymerase to detect a fluorescent reporter signal generated during Real-Time PCR reactions. Amplification and detection were performed using a real-time PCR system (Applied Biosystems^®^ Steponeplus™ Real-time PCR system, Life Technologies™, USA).

### Statistical Analysis

The normality of each variable was initially tested with the Kolmogorov-Smirnov test. Post-race creatine kinase and myoglobin concentration were the only variables that did not follow a normal distribution and thus were analyzed with non-parametric statistics. The comparison between groups (CC *vs* CA) in the variables measured once during the experiment was performed using Student’s t test for independent samples. For the variables measured twice or more during the experiment, the comparison between groups was performed using a two-way ANOVA (group × time). For the non-parametric variables the U-Mann-Whitney test was used for comparison between groups. The data were analyzed with the statistical package SPSS version 20.0 (SPSS Inc., Chicago, IL). The significance level was set at *P* < 0.05. Data are presented as mean ± SD for each group of marathoners.

## Results

**Fig 1:**
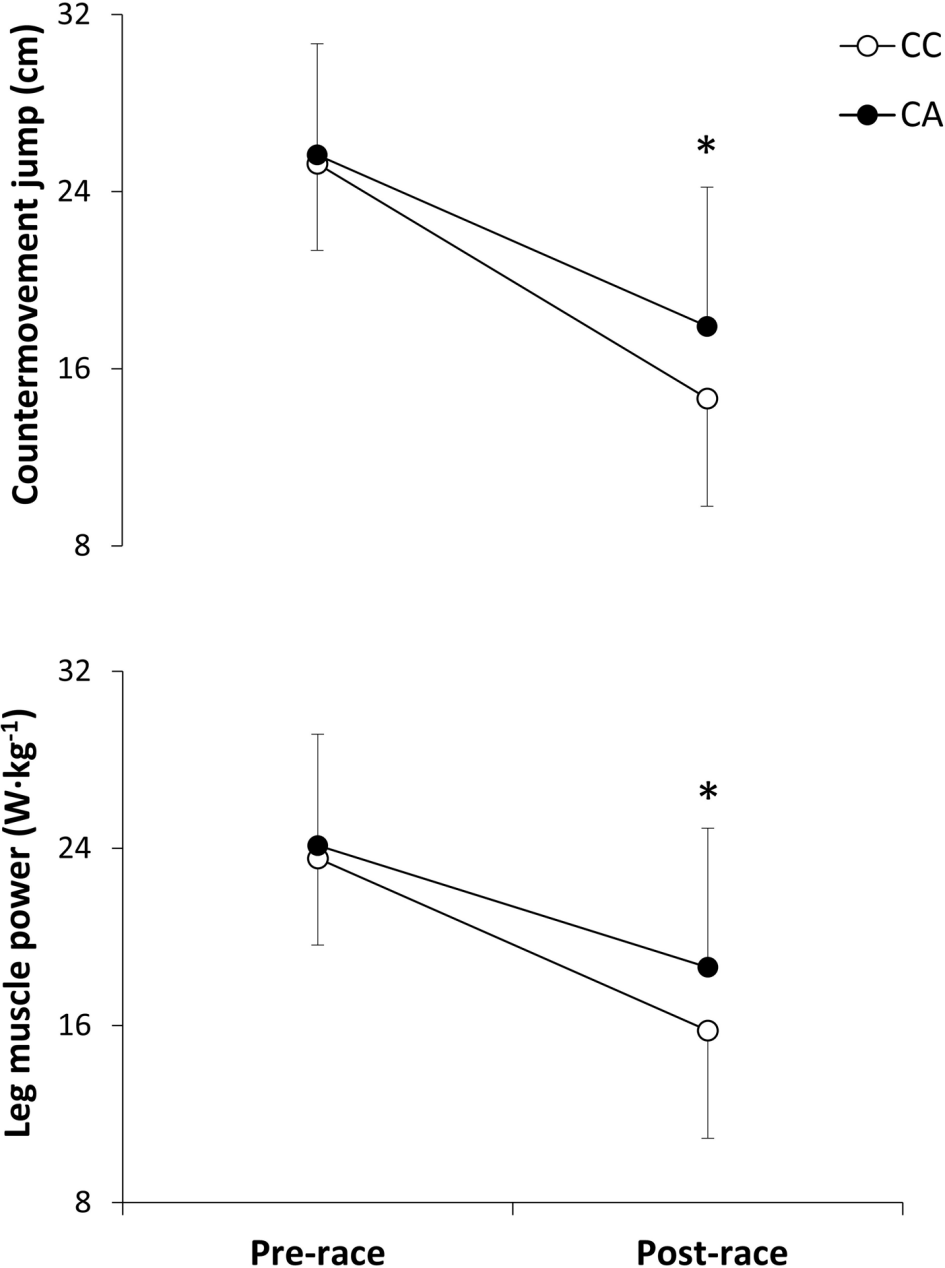
Countermovement jump height and leg muscle power production before and after a marathon competition in runners with different MLCK genotypes (1 SNP tested C37885A). Data are mean ± SD for CC homozygotes and for CA heterozygotes. (*) Different from CC homozygotes, *P* < 0.05.

From the total, 59 participants (88.1% of the sample) were CC homozygotes and 8 participants (11.9%) were CA heterozygotes (Table 1). There were no between-group differences for age, body mass, body height, best race time in the marathon or training routines (*P* > 0.05). Total race time was very similar for CC and CA (228 ± 33 and 234 ± 39 min; *P* = 0.30). Running pace was similar during the whole race between groups and there were no differences in running pace during any 5-km interval. From similar pre-race values, both countermovement jump height and leg muscle power were significantly lower in the CC group than in the CA group (Fig 1; *P* < 0.05) while the reductions in these variables were also higher in the CC group than in the CA group (*P* < 0.05). There were no between-group differences in the levels of perceived exertion (15 ± 2 vs 16 ± 2 A.U.; *P* = 0.21) and muscle pain (6.2 ± 1.7 vs 6.6 ± 1.8 cm; *P* = 0.29) for CC and CA participants, respectively.

**Fig 2:**
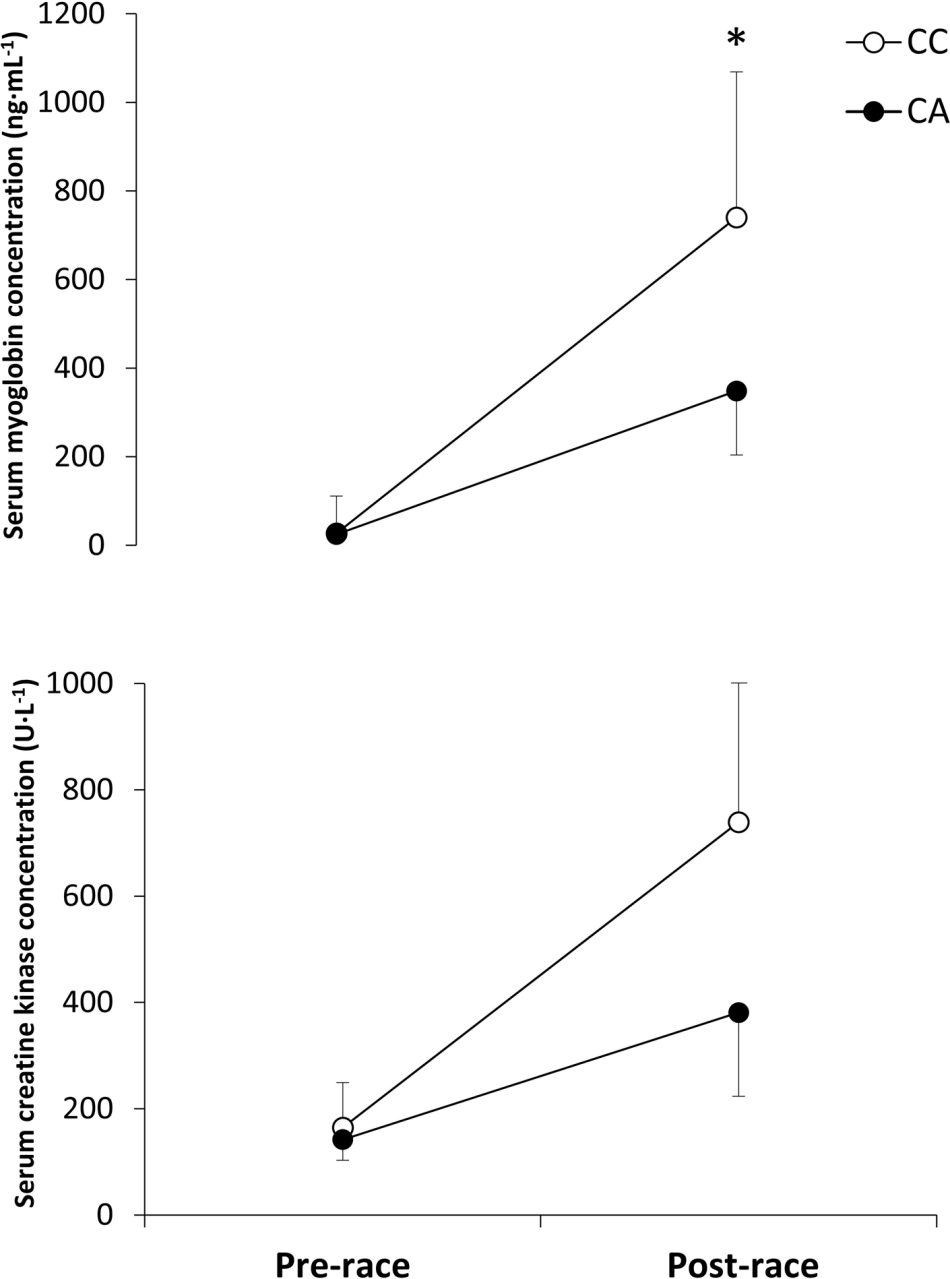
Serum myoglobin and creatine kinase concentration before and just after a marathon competition in runners with different MLCK genotypes (1 SNP tested C37885A). Data are mean ± SD for CC homozygotes and for CA heterozygotes. (*) Different from CC homozygotes, *P* < 0.05.

Fig 2 depicts pre-to-post-competition changes in serum myoglobin and creatine kinase concentrations. From comparable pre-race values, CC homozygotes presented higher post-race serum myoglobin concentrations (*P* = 0.03) while the increase in this variable was also higher than in CA heterozygotes (*P* = 0.04). Nevertheless, post-race serum creatine kinase concentrations (*P* = 0.46) or the pre-to-post-race increase in this variable (*P* = 0.77) were not statistically significant between groups.

## Discussion

The aim of this investigation was to determine the influence of the polymorphic variants of the MLCK C37885A on the development of exercise-induced muscle damage during a marathon competition. The main outcomes of this investigation were: a) amateur runners with CC homozygosis presented higher muscle performance decrements after the marathon than CA heterozygotes, as measured by pre-to-post-race changes in leg muscle power production and jump height during a countermovement jump (Fig 1); CC homozygotes also showed higher post-race serum myoglobin concentrations than CA heterozygotes (Fig 2) although serum creatine kinase concentration was not different between groups at the end of the race; c) however, there were no between-group differences in the self-reported values of muscle pain or perceived exertion while both groups for runners maintained a similar steady-state running pace during the race. These results indicate that the presence of CC homozygosis for MLCK C37885A might predispose amateur runners to higher levels of exercise-induced muscle damage. However, this increased predisposition was resolved without any clinical consequences because no participant in this investigation developed an episode of exertional rhabdomyolysis.

Exertional rhabdomyolysis is a condition in which strenuous exercise or vigorous muscle activity results in ultrastructural damage to the skeletal muscle and it has been identified in several endurance exercise modalities [8, 9]. This exercise-induced muscle damage is characterized by a reduced capacity to generate force, delayed-onset muscle soreness and increases in intramuscular proteins in the blood stream [10, 11]. Although exertional rhabdomyolysis is resolved without serious medical complications in most exercise and sport situations, it can become clinically relevant when muscle proteins–mainly myoglobin- precipitate into the kidneys because this protein can cause acute renal failure, especially when intense exercise is accompanied by heat stress and/or dehydration [5, 12]. It has been suggested that the physical challenges necessary to complete a marathon race might be related to the development of exertional rhabdomyolysis in amateur runners [13] because of the muscle fiber breakdown as the result of the concentric and eccentric muscle actions, combined with the hyperthermia [9] and body water deficit–dehydration- found in most marathoners [14]. Interestingly, the inter-individual level of muscle damage attained during a competitive marathon is highly variable and cannot be explained by factors such as age or previous training status [9].

The explanation as to why some individuals, and not others, incur severe exertion-induced muscle damage during a marathon is still unknown, although it could be related to the variations in genes encoding for specific myofibrillar proteins. While several genes, such as the CKMM gene and the ACTN3 gene, might be related to the ability of the skeletal muscle to resist muscle strain due to the functionality of the proteins they encode [15], the MLCK gene is a good candidate because of the role of the MLCK protein in the development of force by phosphorylation of the RLC of myosin. Childers and McDonald (3] incubated skinned type II fibers with MLCK in rat psoas muscle and found a significant increase in Ca^2+^ sensitivity that ultimately produced greater force and power. Besides, the muscle fibers incubated with MLCK also showed greater force deficits during a series of stretches. Therefore, the MLCK protein and thus the MLCK gene might play a double role during skeletal muscle contraction by increasing force production but also affecting muscle fatigability that can be translated into reduced capacity to endure muscle strain during prolonged exercise routines.

Two previous investigations suggested that the A allele of the MLCK C37885A was associated with an increased likelihood of suffering exercise-induced muscle damage [5, 6]. However, the outcomes of these investigations are difficult to interpret because the type of exercise used to induce muscle damage (elbow flexions in the dominant arm [5] or not described [6]) and the method to evaluate the presence of exercise induced muscle damage (increased creatine kinase activity 4 days after exercise [5] or the existence of a clinical episode of exertional rhabdomyolysis [6]) are difficult to apply to any real exercise or sport scenario. The current investigation used an ecological approximation, because all participants competed in a marathon, an ecological context in which the presence of exercise-induced muscle damage has been repeatedly catalogued [9, 14, 16–18]. In this case, we found that CC homozygotes for C37885A were the runners with the highest intramuscular protein leakage and greater muscle power loss, at least when compared to CA heterozygotes. Despite the statistical differences present between CA and CC marathoners in the variables related to exercise-induced muscle damage, the absence of any individual with an episode of exertional rhabdomyolysis together with the high intra-group variability in the physiological responses after the marathon suggest that the influence of the MLCK C37885A on the development of exercise-induced muscle damage during exercise is limited. It is likely that additional genes encoding other intramuscular proteins also play a role in the development of exercise induced muscle damage.

Our outcomes are contrary to previous research on this topic [5, 6]–and even our initial hypothesis- because we did not find that the A allele for the MLCK C37885A polymorphism was associated to increased levels of exercise induced muscle damage after the marathon. Still, the explanation for these discrepancies is not evident from our data. First, the individuals’ frequency for the most common genotypic variations in MLCK C37885A was similar in the current investigation (88.1% of the marathoners were CC homozygotes and 11.9% were CA heterozygotes) to the ones reported by Clarkson et al. [5] (77.1 / 22.3% for CC and CA individuals, respectively) and by Deuster et al. [6] (78.4 / 19.3% for CC and CA individuals, respectively). These two previous investigations reported a frequency of 0.6-to-2.3% of individuals homozygous for the rare MLCK 37885A allele, while there was no subject with this genotype in our investigation. In any case, the absence of AA homozygotes for the investigated SNP did not explain the differences between CC and CA found in our investigation. Second, Clarkson et al. [5] pointed out that the different levels of exercise induced muscle damage found between CC and CA individuals disappeared when the analysis was adjusted for baseline strength values, indicating that some of the effect attributed to the MLCK genotypic variations were in fact the product of different strength values. In our investigation leg muscle power and countermovement jump height were very similar and thus our outcomes regarding exercise-induced muscle damage are unrelated to between-groups differences in strength or power. Third, while it has been established that phosphorylation of RLC via MLCK plays an essential role in the development of muscle force [1] and it likely produces higher values of contraction-induced muscle damage [3], there is no information to determine whether the increased phosphorylation of RLC is the result of the A or the C allele for the MLCK C37885A. The lack of scientific information on this topic warrants further investigation.

The present investigation also presents some limitations derived from the experimental design selected. Because this study was carried out in a real competitive marathon, factors such as age, training routines and running intensity during the race were not controlled. Nevertheless, we recorded this information and based on the absence of between-group differences (Table 1) we believe that these factors had a negligible influence on the outcomes of this investigation. Besides, it is well established that some of the symptoms related to exertional rhabdomyolysis and exercise-induced muscle damage–such as local muscle pain, muscle power and strength losses and leakage of myocellular proteins into the bloodstream- peak at 24-to-48 h after the end of the damaging exercise [13]. Our experimental protocol included these same physiological variables but measured just after the end of the marathon, while we have no information about the recovery phase after the marathon. Thus, it would be necessary to investigate whether the influence of the MLCK gene is also present during the recovery phase of the exercise-induced muscle damage.

In summary, CC homozygous marathon runners for MLCK C37885A showed higher levels of exertional muscle damage than CA heterozygotes, as measured by higher values of leg muscle power reduction and greater leakage of myoglobin into the circulation. Thus, the genetic variation in the MLCK gene might influence the level of muscle damage attained during endurance running. Because the MLCK phosphorylates the RLC of myosin, predominantly in type II muscle fibers, it is likely that the greater levels of exercise-induced muscle damage found in CC homozygotes are related to the ultrastructural damage in the sarcolemma of these types of fibers during the race. In any case, there was no clinical episode of exertional rhabdomyolysis during the competition that suggests that the higher predisposition of CC homozygous for increased muscle damage during endurance running did not directly translate into exertional rhabdomyolysis, at least during a marathon competition.
