# Correction: Myosin Light Chain Kinase (MLCK) Gene Influences Exercise Induced Muscle Damage during a Competitive Marathon

**DOI:** 10.1371/journal.pone.0168309

**Published:** 2016-12-09

**Authors:** Juan Del Coso, Marjorie Valero, Beatriz Lara, Juan José Salinero, César Gallo-Salazar, Francisco Areces

There is an error in the last sentence of the Abstract. The correct sentence is: CA heterozygotes for MLCK C37885A might present lower exercise-induced muscle damage after a marathon competition than CC counterparts.
